# GLIPR2: a potential biomarker and therapeutic target unveiled – Insights from extensive pan-cancer analyses, with a spotlight on lung adenocarcinoma

**DOI:** 10.3389/fimmu.2024.1280525

**Published:** 2024-02-26

**Authors:** Wei Lin, Siming Zhang, Chunyan Gu, Haixia Zhu, Yuan Liu

**Affiliations:** ^1^ Cancer Research Center Nantong, Affiliated Tumor Hospital of Nantong University, Nantong, China; ^2^ Jiangsu Key Laboratory of Neuropsychiatric Diseases and Institute of Neuroscience, Soochow University, Suzhou, China; ^3^ Department of Pathology, Affiliated Nantong Hospital 3 of Nantong University (Nantong Third People’s Hospital), Nantong, China

**Keywords:** pan-cancer analysis, GLIPR2, LUAD, tumor suppressor, immune infiltration

## Abstract

**Background:**

Glioma pathogenesis related-2 (GLIPR2), an emerging Golgi membrane protein implicated in autophagy, has received limited attention in current scholarly discourse.

**Methods:**

Leveraging extensive datasets, including The Cancer Genome Atlas (TCGA), Genotype Tissue Expression (GTEx), Human Protein Atlas (HPA), and Clinical Proteomic Tumor Analysis Consortium (CPTAC), we conducted a comprehensive investigation into GLIPR2 expression across diverse human malignancies. Utilizing UALCAN, OncoDB, MEXPRESS and cBioPortal databases, we scrutinized GLIPR2 mutation patterns and methylation landscapes. The integration of bulk and single-cell RNA sequencing facilitated elucidation of relationships among cellular heterogeneity, immune infiltration, and GLIPR2 levels in pan-cancer. Employing ROC and KM analyses, we unveiled the diagnostic and prognostic potential of GLIPR2 across diverse cancers. Immunohistochemistry provided insights into GLIPR2 expression patterns in a multicenter cohort spanning various cancer types. *In vitro* functional experiments, including transwell assays, wound healing analyses, and drug sensitivity testing, were employed to delineate the tumor suppressive role of GLIPR2.

**Results:**

GLIPR2 expression was significantly reduced in neoplastic tissues compared to its prevalence in healthy tissues. Copy number variations (CNV) and alterations in methylation patterns exhibited discernible correlations with GLIPR2 expression within tumor tissues. Moreover, GLIPR2 demonstrated diagnostic and prognostic implications, showing pronounced associations with the expression profiles of numerous immune checkpoint genes and the relative abundance of immune cells in the neoplastic microenvironment. This multifaceted influence was evident across various cancer types, with lung adenocarcinoma (LUAD) being particularly prominent. Notably, patients with LUAD exhibited a significant decrease in GLIPR2 expression within practical clinical settings. Elevated GLIPR2 expression correlated with improved prognostic outcomes specifically in LUAD. Following radiotherapy, LUAD cases displayed an increased presence of GLIPR2^+^ infiltrating cellular constituents, indicating a notable correlation with heightened sensitivity to radiation-induced therapeutic modalities. A battery of experiments validated the functional role of GLIPR2 in suppressing the malignant phenotype and enhancing treatment sensitivity.

**Conclusion:**

In pan-cancer, particularly in LUAD, GLIPR2 emerges as a promising novel biomarker and tumor suppressor. Its involvement in immune cell infiltration suggests potential as an immunotherapeutic target.

## 1 Introduction

Cancer constitutes a significant contributor to global mortality and the profound compromise of well-being, exerting its impact on a universal scale (1). Presently, the absence of a comprehensive remedy for cancer is notably conspicuous. The year 2020 bore witness to the encroachment of Coronavirus Disease 2019 (COVID-19), resulting in considerable impediments to both the diagnosis and management of cancer (2). As a concrete illustration, the restriction of healthcare access consequent to the closure of medical facilities precipitated setbacks in the identification and treatment of malignant conditions. These setbacks, in turn, led to a transient decline in cancer incidence, succeeded by a subsequent upsurge in disease progression, culminating in escalated mortality rates. Despite substantial advancements in the sphere of oncological intervention, including immunotherapy, precision-targeted therapy, and radiation therapy (3–5), the 5-year overall survival (OS) rate for afflicted patients persistently eludes attainment of satisfactory levels.

Recent years have witnessed a revolutionary transformation in cancer research with the emergence of high-throughput sequencing technologies and comprehensive molecular analyses (6, 7). These innovations have brought to light novel biomarkers and therapeutic targets with the potential to profoundly impact the realms of cancer diagnosis, prognosis, and treatment strategies. Amid these emerging contenders, glioma pathogenesis related-2 (GLIPR2) has ascended in significance as a hub gene, owing to its multifaceted involvement across diverse domains of disease biology (8, 9).

GLIPR2, also recognized as Golgi-associated plant pathogenesis-related protein 1 (GAPR1), stands as a multifunctional protein that has garnered escalating attention due to its dual engagement in both normal cellular processes and the intricacies of cancer biology. GLIPR2 has been associated with a spectrum of cellular functions encompassing the regulation of autophagy and its entwinement in various neoplastic conditions (10, 11).

To comprehensively elucidate the functional and clinical implications of GLIPR2 across diverse cancer subtypes, this investigation integrates a multitude of analytical methodologies. Differential expression analysis, diagnostic curve evaluation, mutation scrutiny, methylation analysis, and examination of immune infiltration collectively depict the pivotal role of GLIPR2 in cancer pathogenesis. Moreover, validation of the discerned findings through scrutiny of a cohort of non-small cell lung cancer (NSCLC) patients from Nantong Tumor Hospital augments the clinical pertinence of the study. Finally, several lines of experiments indicated the tumor-suppressor function of GLIPR2 in suppressing malignant phenotype and facilitating the sensitivity of treatments. By amalgamating disparate datasets and deploying an array of bioinformatics techniques, this inquiry aspires to unravel the intricate interplay between the dysregulation of GLIPR2 and the evolution of malignancies. Furthermore, by illuminating the molecular mechanisms underpinning its participation and its potential as a diagnostic, prognostic, and therapeutic target, this exploration contributes to a heightened comprehension of the intricacies of cancer biology. It also charts a course for the formulation of precision medicine approaches.

## 2 Methods and materials

### 2.1 Data collection and preprocessing for pan-cancer patients

RNA sequencing data, along with survival information and clinical phenotypic characteristics, were gathered from The Cancer Genome Atlas (TCGA) repository (https://www.cancer.gov/ccg/research/genome-sequencing/tcga), housed within the University of California Santa Cruz (UCSC) Xena platform (http://www.genome.ucsc.edu/). We utilized the STAR (Spliced Transcripts Alignment to a Reference) pipeline to process the RNAseq data, extracting transcripts per million (TPM) values for downstream analysis. Our data filtering strategy involved removing samples lacking clinical information and the exclusion of duplicate entries to ensure the integrity and reliability of the dataset. Following these filtering criteria, we performed data normalization using the log_2_ transformation of the TPM values, with the addition of one to accommodate zero values (log_2_(value+1)). After thorough data refinement and normalization procedures, a comprehensive cohort comprising 10,924 samples of malignant tumor tissues and 727 samples of adjacent paracancerous tissues was assembled for analysis. Simultaneously, non-neoplastic control tissues sourced from the Genotype Tissue Expression (GTEx) project (https://www.gtexportal.org) were procured to complement the dataset. Furthermore, a subset encompassing 301 patients with NSCLC, all of whom possessed pertinent clinical records related to their survival durations, was curated from the clinical archives of the Affiliated Tumor Hospital of Nantong University. Lastly, 18 paired samples (cervical squamous cell carcinoma and endocervical adenocarcinoma: CESC, lung adenocarcinoma: LUAD; lung squamous cell carcinoma: LUSC) from Nantong third hospital were included to describe the expression of GLIPR2. Written informed consent was obtained from each participating patient in this study. The ethics committee of the Affiliated Tumor Hospital of Nantong University and Nantong Third People’s Hospital approved this study.

### 2.2 Expression analysis of GLIPR2

The architectural conformation and subcellular localization (A-431, U-251MG and U20S cell lines) of GLIPR2 were inferred from data accessible in the Human Protein Altas (HPA) repository (https://www.proteinatlas.org/). To elucidate GLIPR2 RNA expression profiles, we harnessed the integrated resources of TCGA (https://www.cancer.gov/ccg/research/genome-sequencing/tcga), coupled with the GTEx consortium, and employed TIMER2.0 (http://timer.cistrome.org/) as a complementary resource. Transforming the expression data through a logarithmic base 2 conversion, we subjected the resultant values to t-tests. Statistical significance was established at a threshold of *P* < 0.05, delineating distinctions in expression patterns between malignant and healthy tissue contexts. Computational analysis was executed employing the R programming language (Version R4.2.1), while the visualization of data distributions was facilitated by means of the “ggpubr” package integrated within the R environment. Furthermore, the HPA repository in conjunction with the Clinical Proteomic Tumor Analysis Consortium (CPTAC) database (https://ualcan.path.uab.edu/) were harnessed to scrutinize the abundance and localization of GLIPR2 at the protein level.

### 2.3 Diagnostic analysis of GLIPR2

In the diagnostic analysis of GLIPR2, we leveraged data from XENA database to assess the potential applicability of GLIPR2 in cancer diagnostics. The evaluation involved the construction of receiver operating characteristic (ROC) curves, aiming to discern the area under the curve (AUC) values. Notably, an AUC exceeding 0.5 indicates substantial diagnostic efficacy. The ROC curve analysis was performed using the Xiantao Academic Online Tool (https://www.xiantaozi.com/), which integrates data from the XENA database processed through the Toil pipeline. This approach unifies samples from the GTEx project with cancer tissue samples from TCGA.

### 2.4 Copy number variation and methylation analysis of GLIPR2

The investigation of distinct neoplastic contexts has involved a thorough examination of the mutational spectra inherent to GLIPR2. To achieve this objective, the computational framework provided by the cBioPortal tool (http://www.cbioportal.org/) was utilized. The initiation of the analytical process entailed the input of “GLIPR2” within the “Query” module, facilitating interaction with the extensive dataset known as the “TCGA Pan Cancer Atlas Studies” cohort. The interplay between the pertinent genetic locus and the various malignancies within this dataset reveals nuanced insights. Through the “cancer type summary” and “mutation” modules, a comprehensive depiction of GLIPR2 genomic perturbations emerges, elucidating intricate details regarding their spatial distribution, typological attributes, and numerical prevalence.

The assessment of methylation status in GLIPR2 across diverse cancer types and their corresponding adjacent tissues was conducted using the UALCAN repository (http://ualcan.path.uab.edu/analysis.html). Changes in DNA methylation profiles could impact gene expression, with regulation primarily influenced by methylation of CpG sites proximal to the promoters (12). To ascertain differentially methylated promoter regions, MEXPRESS was employed to calculate the association between GLIPR2 expression and DNA methylation (https://mexpress.ugent.be/).

### 2.5 Immune infiltration analysis of GLIPR2

The ESTIMATE algorithm was employed to analyze the disparity in stromal score and immune score utilizing the package “estimate” (Version R4.2.1) (13). The examination of the associations between GLIPR2 expression and the tumor mutation burden (TMB), as well as homologous recombination deficiency (HRD), across distinct tumors sourced from TCGA cohorts, was conducted through the Sanger Box platform. Pearson’s rank correlation test was executed, yielding both the partial correlation (cor) and corresponding *p*-value. Explorations into the connections between GLIPR2 expression and immunomodulatory genes, alongside tumor-infiltrating immune cells (TIICs) across multiple tumors. These immune cells encompass B cells, CD4^+^ T memory cells, CD8^+^ T cells, NK cells, monocytes, macrophages, neutrophils, among others. Subsequently, a series of algorithms were formulated to quantify the extent of TIICs infiltration within the tumor microenvironment (TME), leveraging bulk RNA-seq data. However, diverse algorithms and marker gene sets related to TIICs may engender calculation discrepancies. In order to circumvent these inconsistencies, we carried out a comprehensive determination of TIICs infiltration levels using six distinct independent algorithms: CIBERSORT (14), MCP-counter (15), EPIC (16), quanTIseq (17), XCELL (18), and TIMER (19).

### 2.6 Immunotherapy alone and combined with single-cell sequencing cohorts

The cohorts designated for immunotherapy, both in isolation and in conjunction with single-cell sequencing, were retrieved from authoritative databases. Immunotherapy cohorts were sourced from the BEST database (https://rookieutopia.com/appdirect/BEST/) (20). Single-cell expression profiles subsequent to immunotherapeutic interventions were procured from the TISCH database (http://tisch.comp-genomics.org/).

### 2.7 Tissue microarray construction and immunohistochemistry

The real-world cohort study utilized a tumor and paracancer tissue microarray (TMA) obtained from the Affiliated Tumor Hospital of Nantong University (**Supplementary Table S1**). The construction process of the TMA has been previously described (21). For the immunohistochemical (IHC) staining, the primary anti-GLIPR2 antibody (1:20, SantaCruz Biotechnology, sc-398529, USA) was employed. Following three washes with phosphate-buffered saline (PBS), the tissue sections were incubated with a secondary antibody (Poly-HRP-goat-anti-mouse antibody) for 20 minutes at 37°C, followed by staining with a diaminobenzidine solution. Subsequently, the TMA slides were scanned using the Nikon microscopy system (Japan). The labeling intensity was estimated as negative (0), weak (1), moderate (2) or strong (3). The extent of staining, defined as the percentage of positively stained cells, was scored as 1 (≤10%), 2 (11−50%), 3 (51−80%) and 4 (>80%). The total immunoreactive score (IRS) was obtained by multiplying the staining intensity score and the staining extent score and ranked from 0 to 12 (22, 23). The evaluation of staining intensity was carried out by two pathologists independently, who were kept blinded to the associated clinical data.

### 2.8 Cell culture and plasmid transfection

Beas-2b, H1299, and PC9 cells were cultured in RPMI 1640 medium supplemented with 10% fetal bovine serum (Gibco, Grand Island, NY, USA), and 1% Penicillin-Streptomycin (NCM Biotech, China). Similarly, A549 cells were nurtured in F-12K medium with 10% FBS. The maintenance of all cell lines in their respective culture media ensured optimal growth and experimental conditions. Additionally, regular screening for Mycoplasma species was conducted prior to any experimental procedures.

The plasmids for GLIPR2 overexpression and control were constructed by Shanghai Jikai Gene Technology Co. Ltd. Initially, high-fidelity PCR amplification was employed to obtain the GLIPR2 cDNA, which was subsequently inserted into the Age I site of the GV208 plasmid. Following this, the purified plasmid was transformed into competent cells for amplification, followed by plasmid extraction for subsequent use. H1299 cells in the logarithmic growth phase were seeded in six-well plates at a density of 5 × 10^5^ cells per well, with three replicates per group. Upon achieving 60%-70% confluence, H1299 cells were transfected with the plasmid using Lipofectamine 3000 (Life Technologies).

### 2.9 Quantitative real-time PCR

The protocol for RNA extraction and quantitative real-time PCR (qRT-PCR) followed established procedures as outlined in the literature (21). Upon cell thawing, a minimum of three passages was conducted before commencing experimental procedures. Subsequently, cells underwent centrifugation at 12,000 × g and were suspended in TRIzol reagent (Invitrogen, USA) for RNA extraction. RNA purification involved chloroform extraction followed by isopropanol precipitation. Post RNA extraction, concentrations were determined and normalized through dilution processes. A total of 500 ng of RNA was reverse transcribed into cDNA utilizing the M-MLV kit per the manufacturer’s instructions (Accurate Biology, China). QPCR were performed using iQ SYBR green (AG11701, Accurate Biology, China) on a BioRad CFX97 instrument. A standard curve was generated by 1:10 dilutions of a reference cDNA sample to amplify all target PCR products. Transcript abundance was determined by normalization to human GAPDH (Sangon Biotech, China). Experimental samples were compared against this standard curve to ascertain relative transcript abundance. The primer sequences used for GLIPR2 amplification are provided as follows: forward primer, 5′-GAAGATGGGCGTGGGGAAGG-3’; reverse primer, 5′-TTACTTCTTCG GCGGCAGGAC-3’.”.

### 2.10 Immunofluorescence

The immunofluorescence protocol for cellular analysis was conducted in accordance with previously outlined procedures (24). Briefly, the cells underwent a series of procedures including three washes with PBS, fixation with 4% paraformaldehyde for 20 minutes, and treatment with 1% Triton X-100 for 10 minutes. Subsequent to a 1-hour blocking step, the cells were incubated with anti-GLIPR2 antibody sourced from Santa Cruz, diluted to 1:20, and maintained at 4°C for 18 hours. Following this, the cells were exposed to donkey anti-mouse 555 secondary antibody, diluted to 1:500 (Millipore, USA). DAPI staining of the nucleus was conducted for 5 minutes. Finally, high-resolution images of the stained sections were captured using a scanning microscope (Nikon, Japan).

### 2.11 Cell invasion and wound healing assay

H1299 cell invasiveness was assessed utilizing 24-well transwell chambers (8μm, Corning, Lowell, MA, USA). In a succinct sequence, following a 24-hour incubation period, the chambers underwent cleansing with cotton swabs, fixation with 4% paraformaldehyde for 20 minutes, and subsequent staining with crystal violet. The enumeration of cells was conducted in three randomly selected fields within each chamber, and the resultant values were averaged.

For the evaluation of migratory potential, a single-cell suspension was introduced into a 6-well plate and cultivated until cells reached 90%-100% confluency. Subsequently, a controlled and vertical scratch was generated using a 200 µl pipette tip, creating a wound. Detached cells were systematically purged with PBS, and the medium was subsequently replaced with 1 ml of serum-free medium. The 24-well plate was positioned in the Live Cell Imaging System (Leica, Brunswick, Saxony, Germany), capturing images of the wound at both 0 h and 24 h. Measurements of wound distances were taken, and the rate of wound healing was evaluated.

### 2.12 Drug sensitivity analysis

H1299 cells were seeded into 96-well plates at a density of 5*10^3 cells per well. Following plasmid transfection, the cells were exposed to varying concentrations of cisplatin (MedChemExpress, USA) at 12.5, 25, and 50 µM, or subjected to irradiation with X-rays at doses of 2, 4, 6, and 8 Gy, administered at a dose rate of 1 Gy. After 48 hours, cell proliferation was assessed using a colorimetric assay employing the cell counting Kit-8 (CCK-8; Bimake, Houston, TX, USA), following the manufacturer’s instructions.

### 2.13 Statistical analysis

Data processing, statistical analysis, and visualization were comprehensively performed using the R 4.2.1 software package. For datasets exhibiting normal distribution, the unpaired Student t-test was applied, whereas for datasets deviating from normal distribution, the Wilcoxon test was employed. Pearson’s correlation coefficients were utilized to evaluate the association between two continuous variables. Considering the potential impact of skewed data, Spearman’s correlation analysis was also performed to ensure a comprehensive examination of the relationship. The prognostic value was evaluated by Kaplan‐Meier analysis. A significance level of *P* < 0.05 was considered indicative of statistical significance. All reported *p*-values resulting from TCGA data, were subjected to adjustment for multiple testing using the Benjamini-Hochberg procedure to control the false discovery rate (FDR).

## 3 Results

### 3.1 Procedural overview and expression analysis of GLIPR2 in cancer

**Figure 1:**
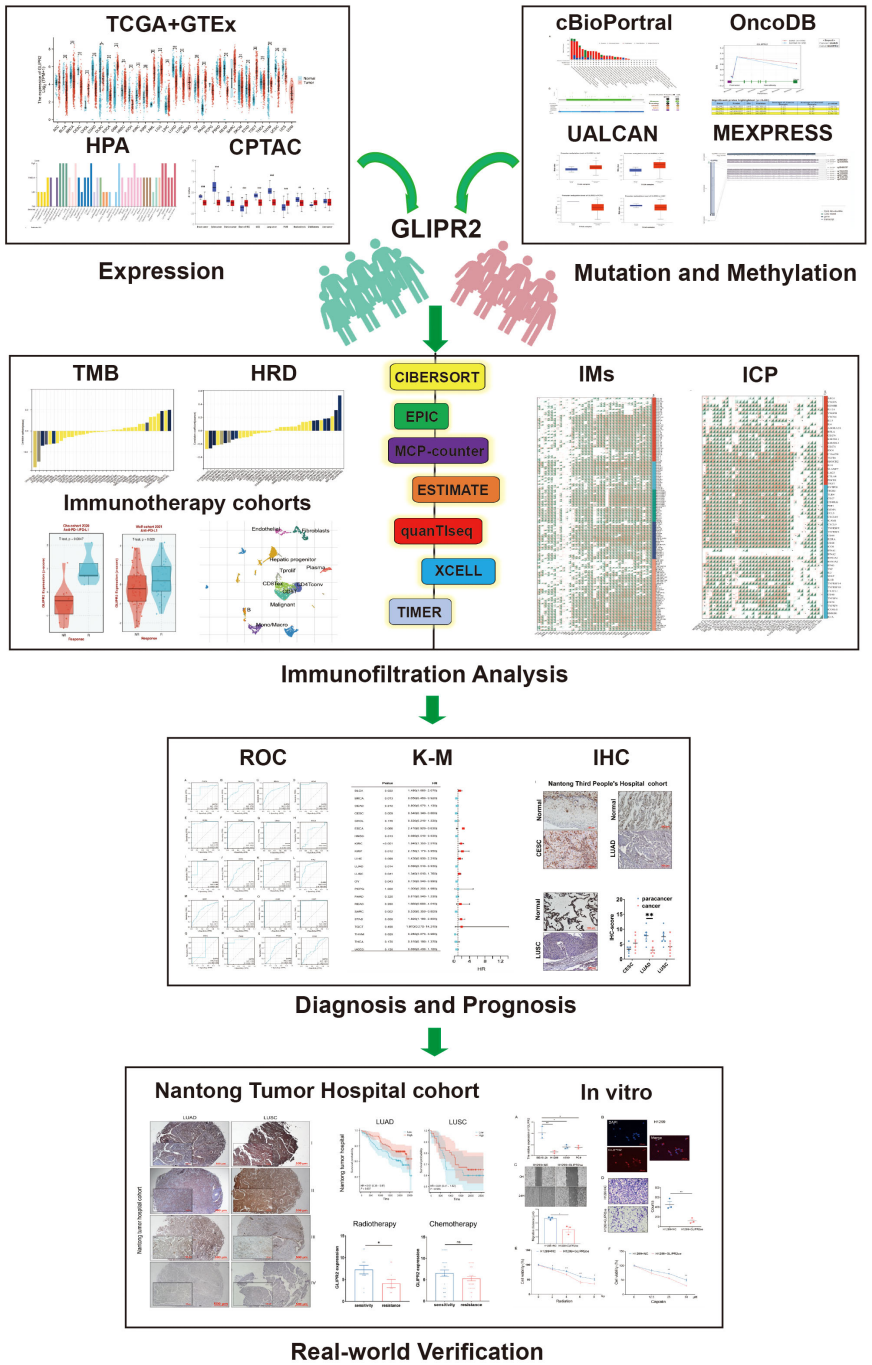
The flow chart of the study.

**Figure 2:**
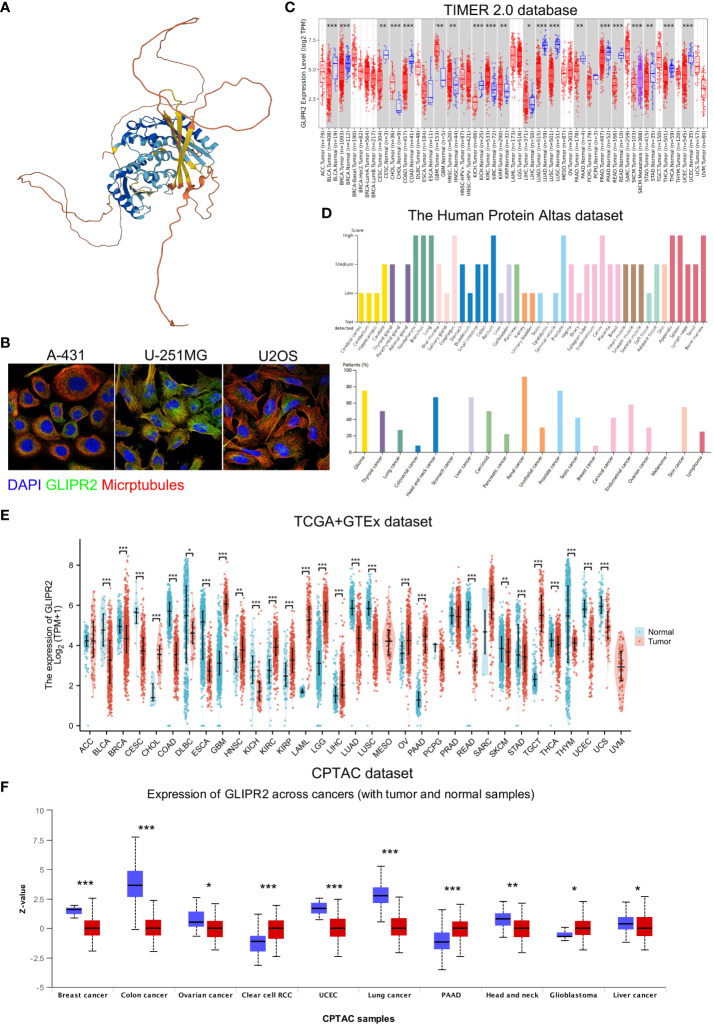
Comprehensive overview of GLIPR2 in cancer. **(A)** Representation of the molecular structure of GLIPR2. **(B)** Visualization of GLIPR2’s subcellular distribution within cells. **(C)** Analysis of GLIPR2 gene expression at the RNA level across diverse cancer types, providing insights into the transcriptomic landscape. **(D)** Examination of GLIPR2 expression at the protein level, showcasing tissue-specific spatial distribution and prevalence among cancer patients. **(E)** Comparative analysis of GLIPR2 expression at the RNA level, highlighting differences between normal and cancerous tissues. **(F)** Comprehensive proteomic analysis depicting GLIPR2 expression across different cancers, complementing the preceding panels with a protein-level perspective. *P < 0.05, **P < 0.01, ***P < 0.001.

The study’s procedural overview is depicted in **Figure 1**. Primarily, the current understanding underscored that the protein structure of GLIPR2 comprised multiple α-folds, as delineated in **Figure 2A**. It is predominantly distributed in the cytoplasm, and intriguingly, its observation in the U-251MG cell line reveals co-localization with microtubule proteins, suggesting its potential involvement in the constitution of the cellular cytoskeleton (**Figure 2B**). Analysis of GLIPR2 gene expression patterns was executed using the TIMER 2.0 database. The derived outcomes revealed a prevailing downregulation of GLIPR2 across a spectrum of cancers (**Figure 2C**), inclusive of but not limited to bladder urothelial carcinoma (BLCA), breast invasive carcinoma (BRCA), colon adenocarcinoma (COAD), kidney chromophobe (KICH), LUAD, LUSC, pancreatic adenocarcinoma (PAAD), prostate adenocarcinoma (PRAD), CESC, and rectum adenocarcinoma (READ). Employing the HPA database, the investigation into the protein expression profile of GLIPR2 across various malignancies transpired. As portrayed in **Figure 2D**, heightened expression of GLIPR2 was conspicuous within tissues like the nasopharynx, bronchus, lung, esophagus, rectum, prostate, cervix, appendix, spleen, and bone marrow. Notably, a discernable trend emerged, where most malignancies exhibited moderate cytoplasmic positivity, whereas colorectal, breast, gastric, and pancreatic cancers displayed a general lack of such positivity. To gain deeper insights into GLIPR2 expression patterns, an exploration encompassing TCGA, GTEx and CPTAC datasets was conducted. These endeavors elucidated an augmented GLIPR2 expression in BLCA, BRCA, cholangiocarcinoma (CHOL), amongst others, in contrast to a diminution in breast, colon, ovarian cancers, among others (**Figures 2E, F**). Cumulatively, these findings intimated that GLIPR2 evinced dysregulation across diverse cancer types, thus postulating its pivotal involvement in the sphere of cancer diagnosis.

### 3.2 Genetic alterations and methylation patterns of GLIPR2 in various cancers

The cBioPortal tool revealed noteworthy variations in the genetic makeup of GLIPR2, exhibiting distinct patterns of alteration frequencies across different malignancies. In the context of acute myeloid leukemia (LAML), a deep deletion event was detected at a prevalence rate of 0.5%. Furthermore, diffuse large B-cell lymphoma (DLBC) demonstrated an amplification frequency of 2.08%, followed by uterine carcinosarcoma (UCS) at 1.75%, ovarian serous cystadenocarcinoma (OV) at 1.03%, skin cutaneous melanoma (SKCM) at 0.9%, testicular germ cell tumors (TGCT) at 0.67%, PRAD at 0.61%, liver hepatocellular carcinoma (LIHC) at 0.27%, and brain lower grade glioma (LGG) at 0.19%. In addition, the identified alterations encompassed diverse combinations of two or more mutational types within other implicated tumor types (**Supplementary Figure S1A**). Notably, within GLIPR2, a total of 34 variants of uncertain significance (VUS) were identified across various tumor contexts (**Supplementary Figure S1B**; **Supplementary Table S2**).

**Figure 3:**
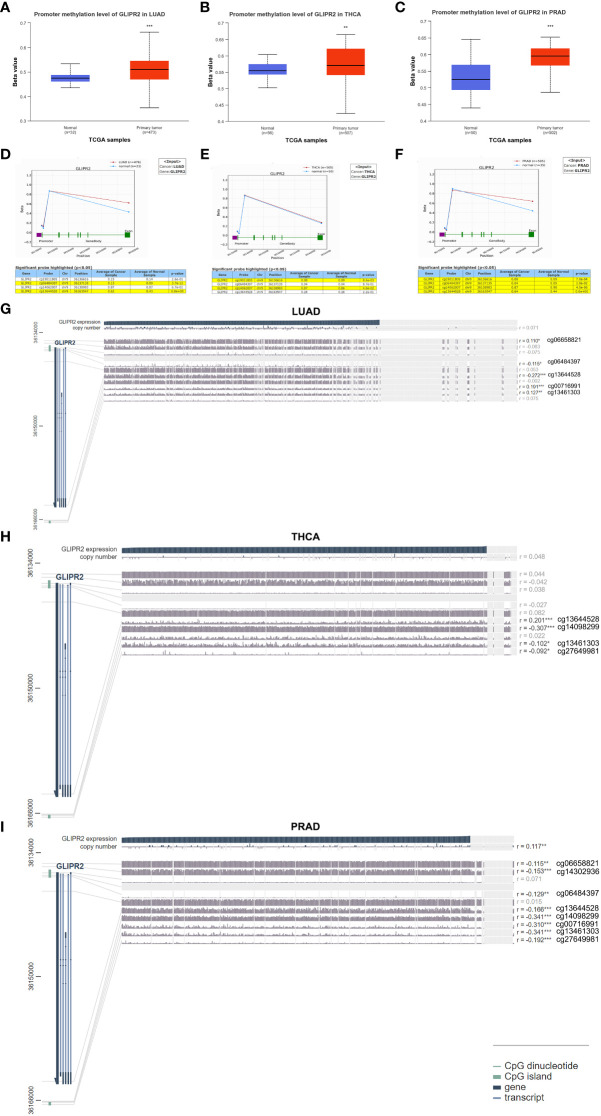
Methylation analysis of GLIPR2. Methylation analysis of GLIPR2 in lung adenocarcinoma (LUAD, **A**), thyroid carcinoma (THCA, **B**), and prostate adenocarcinoma (PRAD, **C**) and normal tissues was conducted using the UALCAN database. Exploration of GLIPR2 methylation status in LUAD **(D)**, THCA **(E)**, and PRAD **(F)** was performed via the OncoDB database. Visualization of the methylation sites within the GLIPR2 DNA sequence associated with gene expression was accomplished using MEXPRESS in LUAD **(G)**, THCA **(H)**, and PRAD **(I)**. The GLIPR2 expression is represented by the blue line. *Pearson*’s correlation coefficients and p-values for methylation sites and query gene expression are provided on the right side. **P < 0.01, ***P < 0.001.

Aberrant DNA methylation patterns are implicated in gene dysregulation in cancer (25). To investigate the causal relationship between aberrant expression patterns of GLIPR2 and methylation, we utilized the UALCAN database (26) along with OncoDB (27) to explore abnormal GLIPR2 methylation patterns in both normal and tumor tissues. Furthermore, we utilized MEXPRESS (28) to examine the correlation between GLIPR2 expression and CpG islands in tumor tissues. By integrating gene methylation differences between cancer and normal groups from the UALCAN database (**Supplementary Figure S2**), we observed that the reduced expression in LUAD, THCA, and PRAD may be associated with increased methylation (**Figures 2C**, **3A–C**). Conversely, in cancers where methylation abnormalities are decreased, such as HNSC, elevated expression appears to synchronize with decreased methylation (**Figure 2C**; **Supplementary Figure S2**).

To further refine the macroscopic dysregulation of methylation expression into microscopic differences at methylation sites, we conducted further validation through OncoDB database. We identified that in comparison to adjacent normal tissues, LUAD exhibited high methylation at the cg06484397 and cg13644528 sites, THCA showed elevated methylation at cg14062007, and PRAD displayed increased methylation at cg13644528 (**Figures 3D–F**). In MEXPRESS database, changes in methylation sites cg06484397 (R = -0.115) and cg13644528 (R= -0.272) in LUAD were negatively correlated with GLIPR2 expression, while in PRAD, methylation at cg13644528 (R= -0.166) showed a negative correlation with GLIPR2 expression (**Figures 3G–I**). These commonalities suggest that targeting cg06484397 and cg13644528 in LUAD, as well as cg13644528 in PRAD, may restore normal GLIPR2 expression levels. Thus, these sites could serve as potential therapeutic targets for gene therapy in LUAD and PRAD.

### 3.3 Advancing immune landscape characterization and immunotherapeutic potential of GLIPR2 in diverse cancers

**Figure 4:**
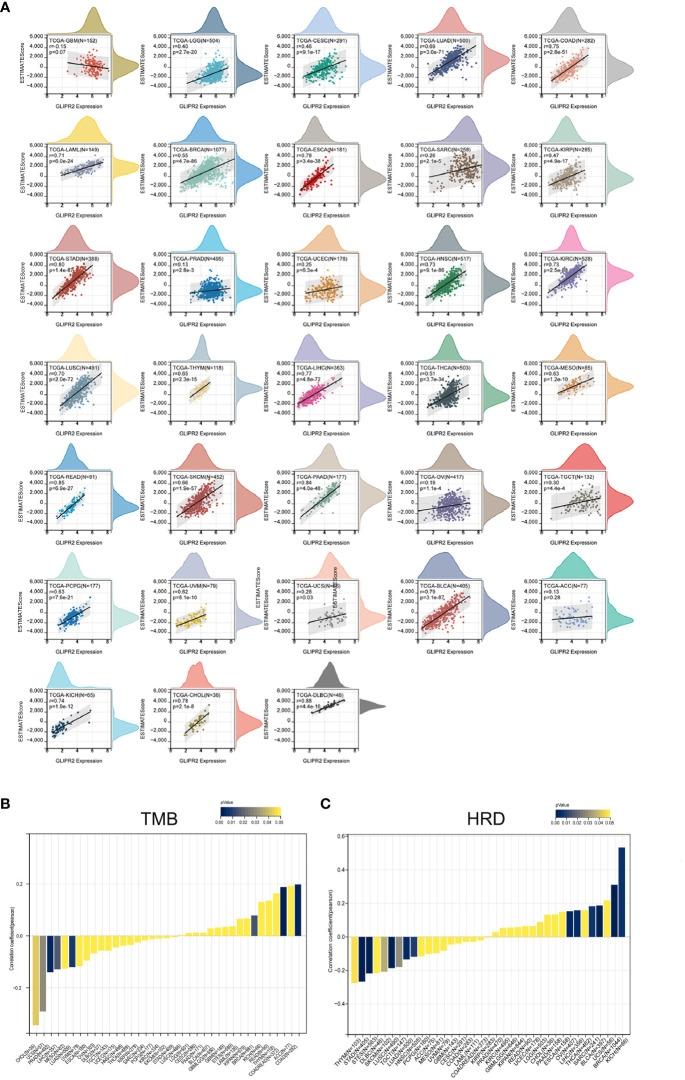
Immune assessment of GLIPR2: estimation, correlation, and association. **(A)** Estimate score. **(B)** Correlation with tumor mutation burden. **(C)** Correlation with homologous recombination repair defects.

The substantial influence exerted by the TME on the progression of cancer is universally acknowledged. Comprising a complex interplay of tumor cells, stromal elements, and immune components, the TME orchestrates intricate and dynamic interactions (29). The Estimation of Stromal and Immune cells in Malignant Tumor tissues using ESTIMATE algorithm have emerged as a robust computational tool for quantifying the infiltration of stromal and immune cells, thereby revealing immune scores and stromal scores. Our investigation into the expression of GLIPR2 has uncovered a positive correlation with immune scores in several cancer types, including low-grade glioma (LGG), CESC, LUAD, as well as other neoplastic tissues. However, it is important to note the observed negative correlation in GBM, although the *p*-value of 0.07 and correlation coefficient (r) of -0.15 suggest that this association may not reach conventional levels of statistical significance (**Figure 4A**).

Employing the metrics of TMB and HRD, the potential of GLIPR2 as an indicator of immunotherapeutic responses across diverse cancer types was ascertained. The examination revealed a positive nexus between GLIPR2 expression and TMB in COAD (*P* < 0.001), READ (*P* < 0.001), and BRCA (*P* = 0.014). Conversely, an inverse relationship transpired in LUAD (*P* = 0.007), PRAD (*P* = 0.002), LIHC (*P* = 0.016), UCS (*P* = 0.029), and CHOL (*P* = 0.043) (**Figure 4B**; **Supplementary Table S3**). Moreover, a positive correlation between GLIPR2 expression and HRD materialized in LGG (*P* = 0.049), BRCA (*P* < 0.001), SARC (*P* = 0.006), LIHC (*P* = 0.003), OV (*P* = 0.002), BLCA (*P* < 0.001), and KICH (*P* = 0.005). In contrast, a negative correlation was discerned in LUAD (*P* = 0.003), stomach and esophageal carcinoma (STES, *P* < 0.001), stomach adenocarcinoma (STAD, *P* < 0.001), HNSC (*P* = 0.008), LUSC (*P* < 0.001), THYM (*P* = 0.048), TGCT (*P* = 0.029), and SKCM (*P* = 0.037) (**Figure 4C**; **Supplementary Table S4**).

**Figure 5:**
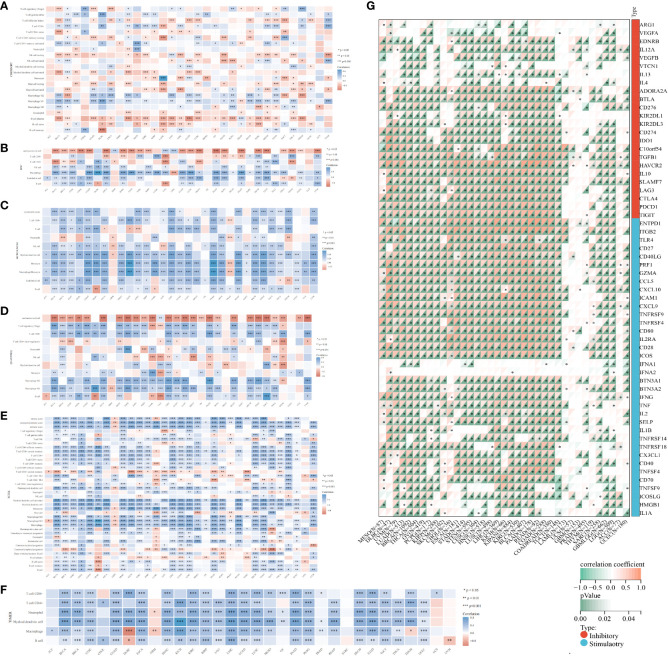
Algorithmic exploration of immune cell infiltration and immunomodulation. Immune cell infiltration was rigorously assessed through a series of mRNA-based immune infiltration prediction algorithms, including CIBERSORT **(A)**, EPIC **(B)**, MCP-counter **(C)**, quanTIseq **(D)**, XCELL **(E)**, and TIMER **(F)**. **(G)** Correlation of GLIPR2 expression levels with immune checkpoint-related genes, darker colors correspond to smaller *p*-values, indicating a higher level of statistical significance in the correlation. *P < 0.05, **P < 0.01, ***P < 0.001.

The TCGA dataset underwent deconvolution through a composite application of computational algorithms, including CIBERSORT, EPIC, MCP-counter, quanTIseq, XCELL, and TIMER (**Figures 5A–F**). The findings underscored substantial disparities in the inferred proportions of distinct cell populations across these algorithmic methodologies. Nonetheless, a consistent pattern emerged in the prevalence of M0 (naïve) macrophages and uncharacterized cellular entities between adjacent and tumor cohorts, aligning coherently across the entire spectrum of accessible techniques. Notably, the neoplastic specimens were conspicuously infiltrated by M1 macrophages and regulatory T (Treg) cells. Of distinct significance, the estimates pertaining to M1 macrophages and Treg cells were uniquely achievable through CIBERSORT, quanTIseq, and XCELL. The resulting revelations collectively unveiled a marked augmentation in the incidence of Treg cells and M1 macrophages within the tumor milieu, except for the CIBERSORT algorithm which indicated a reduction in Treg cell abundance. Concurrently, the abundance of CD8^+^ T cells, estimable through CIBERSORT, MCP-counter, quanTIseq, XCELL, and TIMER algorithms, exhibited a conspicuous elevation within tumor specimens, except for the EPIC estimate which indicated a notable decline. Notably, QuanTIseq emerged as the solitary technique enabling the quantification of cellular fractions, thus facilitating comprehensive comparisons within and between samples. Further elucidation of the statistical significance of inter-algorithmic divergences is provided in **Supplementary Table S5**.

In the context of our comprehensive pan-cancer analysis aimed at deciphering the immunological implications of GLIPR2, the identification of specific malignancies conducive to anti-GLIPR2 immunotherapy holds paramount significance. Our findings elucidated a discernible positive correlation of GLIPR2 with most immunomodulatory elements across various cancers, including kidney papillary cell carcinoma (KIRC), OV, pan-kidney cohort (KIPAN), LIHC, BRCA, LUAD, THCA, PAAD and BLCA (**Supplementary Figure S3**; **Supplementary Tables S6, S7**). Notably, the emergence of immune checkpoint (ICP) blockade proteins as promising candidates for cancer immunotherapy prompted us to conduct a meticulous evaluation of the intricate interplay between GLIPR2 expression levels and the expressions of ICP genes across various malignancies. Remarkably, GLIPR2 exhibited a consistently positive correlation with the expression of ICP genes across various cancers, including LUAD, KIPAN, LIHC, BRCA, THCA, PAAD, KIRC, OV, BLCA (**Figure 5G**; **Supplementary Tables S8, S9**).

**Figure 6:**
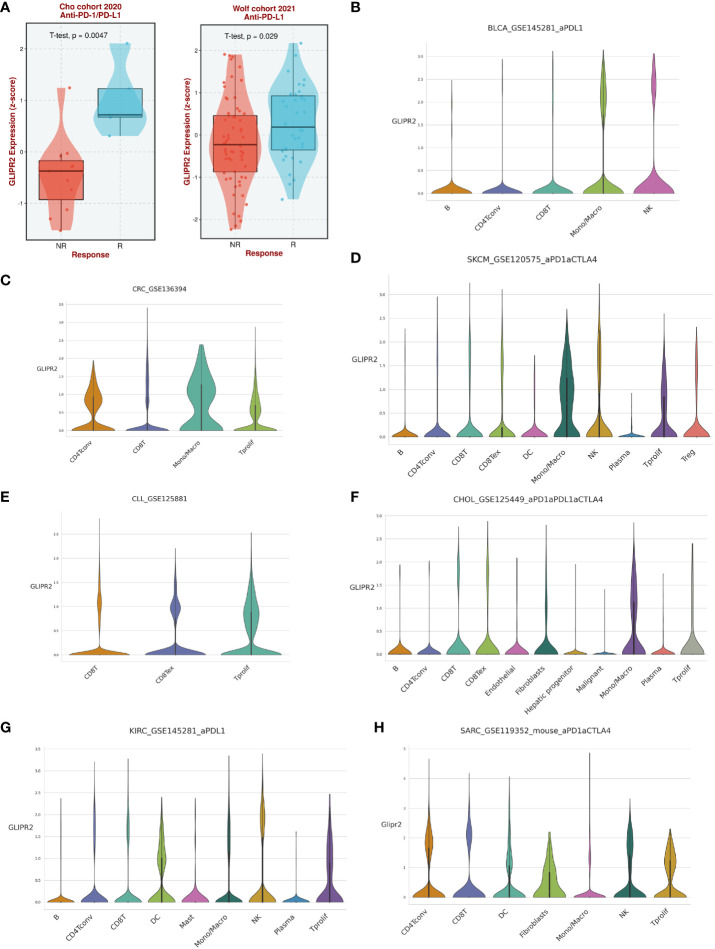
The expression of GLIPR2 predicts a more favorable immunotherapy outcome in patients. **(A)** Patients with high GLIPR2 expression have a better clinical response to immune therapy. **(B**–**H)** Distribution of GLIPR2 expression in different clusters of cancer-resident immune cells at single cell level.

Analysis of immunotherapy cohort data suggests that GLIPR2 expression level is closely related to the patient’s response to immunotherapy (**Figure 6A**). To further reveal the underlying mechanisms, we analyzed the immune cell types in the gene profiles of pan-cancer receiving immunotherapy by single cell sequencing. GLIPR2 expression was found to be enriched in monocyte/macrophage, NK, and T proliferation cells, suggesting potential roles in immune cell recruitment and alterations in the immune microenvironment. Intriguingly, this expression pattern of GLIPR2 was robust to pan-cancer (**Figures 6B–H**).

In concise summation, the prominent role of GLIPR2 in shaping the landscape of immune infiltration across diverse cancers is manifest, firmly positioning it as a compelling candidate for pioneering immunotherapeutic interventions within the realm of oncology.

### 3.4 Deciphering prognostic and diagnostic significance of GLIPR2

**Figure 7:**
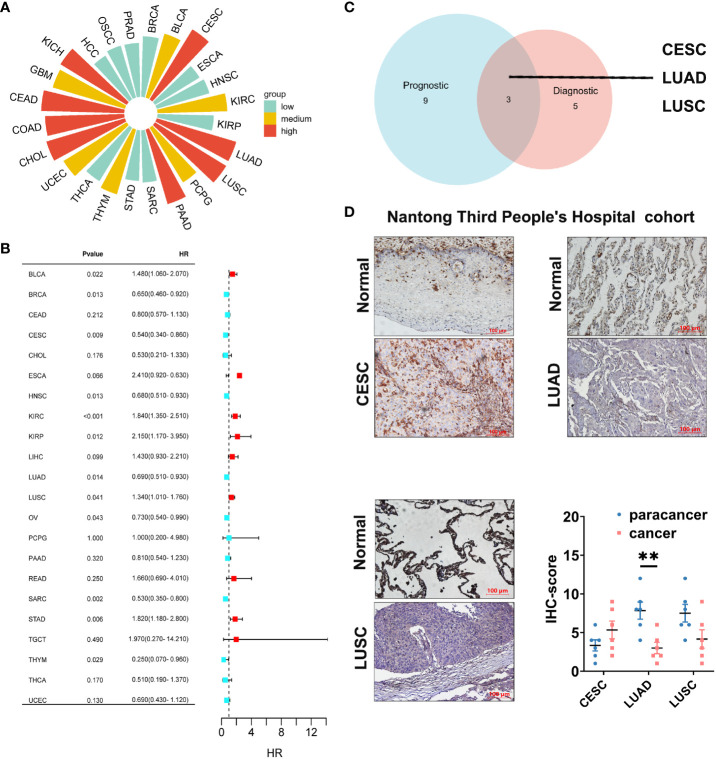
Diagnostic and prognostic value analysis of GLIPR2. **(A)** Prognostic value of GLIPR2 in pan-cancer. **(B)** Prognostic value of GLIPR2 in pan-cancer. **(C)** The intersection of different cancer between the diagnostic and the prognostic value. **(D)** The protein expression of GLIPR2 among CESC, LUAD and LUSC in Nantong Third People’ Hospital cohort, scale bar = 100 μm (n = 6, **P < 0.01).

Cancer diagnosis and prognosis monitoring are critical elements in mitigating cancer-related mortality (30). Markers demonstrating both prognostic and predictive value across diverse cancers warrant meticulous investigation to substantiate their clinical utility. In this study, we employed ROC curves to assess the discriminative potential of GLIPR2 expression levels between malignant and non-neoplastic tissues. In the context of evaluating diagnostic performance, the AUC was selected as the principal metric to measure the discriminative efficacy of our model. In adherence to established conventions, an initial threshold of AUC > 0.5 was employed to delineate performance surpassing random chance. The graphical representation of these ROC curves is illustrated in **Supplementary Figure S4** (**Supplementary Table S10**). The derived AUC values provided compelling evidence that GLIPR2 exhibited a robust capacity to effectively discriminate between malignancy and normalcy across diverse cancer types. However, our primary emphasis is on highlighting exceptional diagnostic accuracy. Consequently, we specifically emphasize instances where the AUC exceeds the threshold of 0.9. Noteworthy observations include CESC (AUC=0.977), CHOL (AUC=0.975), COAD (AUC=0.943), colorectal adenocarcinoma (CEAD, AUC=0.988), KICH (AUC=0.924), LUAD (AUC=0.987), LUSC (AUC=0.994), and PAAD (AUC=0.925), thereby reinforcing the diagnostic potential attributed to GLIPR2 (**Figure 7A**). Subsequently, KM analysis was employed to assess the prognostic value of pan-cancer GLIPR2 levels in patients. In the majority of cancers, such as BRCA, CESC, HNSC, LUAD, OV, SARC and THYM, GLIPR2, acting as a protective factor, demonstrated a reduced risk of death. Conversely, in cancers such as BLCA, KIRC, KIRP, LUSC and STAD, an elevated expression of GLIPR2 was associated with an increased risk of mortality (**Figure 7B**) Integrated prognostic and diagnostic analysis identified LUAD, LUSC, and CESC as cancers most likely to benefit from the GLIPR2 biomarker (**Figure 7C**). Pathological validation revealed a pronounced decrease in GLIPR2 expression in LUAD (**Figure 7D**; **Supplementary Figure S6**).

### 3.5 Predictive merit of GLIPR2 infiltration for NSCLC in a real-world cohort

**Figure 8:**
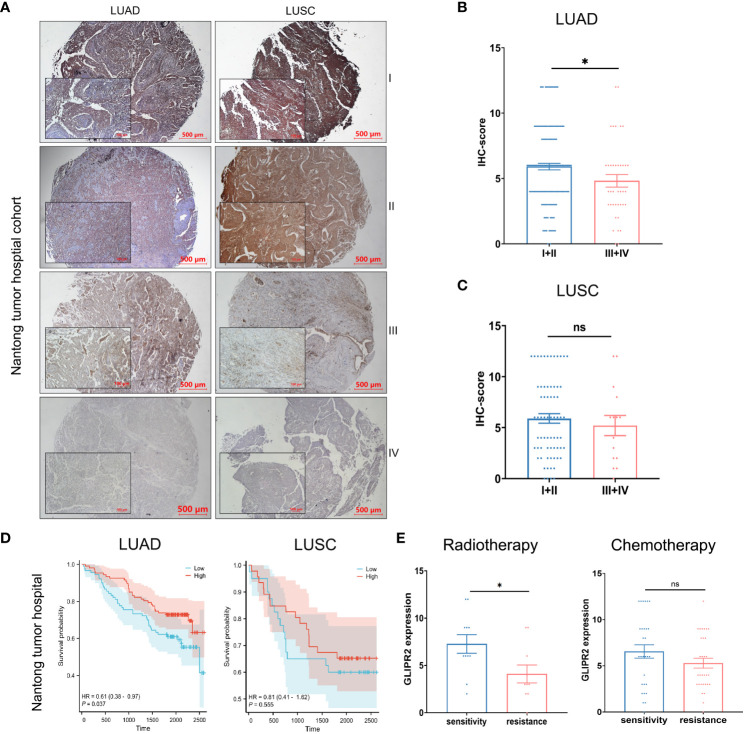
Protein expression and prognostic analysis of GLIPR2 in a real-world cohort. **(A)** Representative expression patterns of GLIPR2 in tumor and peritumor regions, scale bar = 500 μm. **(B, C)** GLIPR2 expression in different stages of LUAD and LUSC, scale bar = 500 μm [**(B)**, n = 194; **(C)**, n = 83]. **(D)** Overall survival (OS) curves according to GLIPR2^+^ infiltration level of patients in Nantong Tumor Hospital cohort. **(E)** The predictive value of GLIPR2 expression was explored in the context of therapeutic interventions, encompassing post-radiotherapy and post-chemotherapy LUAD patients in Nantong Tumor Hospital cohort (Radiotherapy, n = 10-11; Chemotherapy, n = 29-31). *P < 0.05, ”ns“ means ”not significant“.

NSCLC, known for its status as the most prevalent and lethal cancer globally (31), became the primary focus of our investigation following a comprehensive pan-cancer assessment of GLIPR2. Then we embarked on a focused inquiry within a NSCLC cohort sourced from Nantong Tumor Hospital. Notably, within the context of LUAD, IHC scores for GLIPR2 in stage III and IV cases exhibited statistically significant decrease in comparison to stages I and II, a trend that was not evident in LUSC specimens (**Figures 8A–C**).

In consideration of these compelling findings, we undertook meticulous survival analyses predicated upon the levels of GLIPR2^+^ infiltration within the cohorts of LUAD and LUSC patients derived from Nantong Tumor Hospital. Within the realm of LUAD, heightened expression of GLIPR2 was associated with a favorable prognosis, whereas discerning significant survival disparities of GLIPR2 expression was not observed in the context of LUSC (**Figure 8D**). Expanding our investigative scope to encompass the extensively accessible TCGA dataset, we observed congruence between outcomes derived from the Nantong Tumor Hospital cohort and the TCGA dataset (**Supplementary Figure S5**). This concordance substantially bolsters the veracity of our findings on a broader scale.

In an effort to comprehensively gauge the predictive potential of GLIPR2 infiltration density to therapeutic responses, we delved into various treatment modalities. Specifically, we dissected post-radiotherapy LUAD patients, post-chemotherapy LUAD patients, post-radiotherapy LUSC patients, and post-chemotherapy LUSC patients. Notably, amidst post-radiotherapy LUAD patients, those evincing augmented GLIPR2^+^ infiltration levels exhibited correspondingly elevated levels of expression in radiation-sensitive cases in comparison to their radiation-resistant counterparts. This discernment underscores the latent utility of GLIPR2 expression as a predictive biomarker within the context of radiotherapy response within the LUAD patient stratum (**Figure 8E**).

### 3.6 GLIPR2 acts as a tumor suppressor in LUAD, suppress various malignant phenotypes of H1299 cells *in vitro*

**Figure 9:**
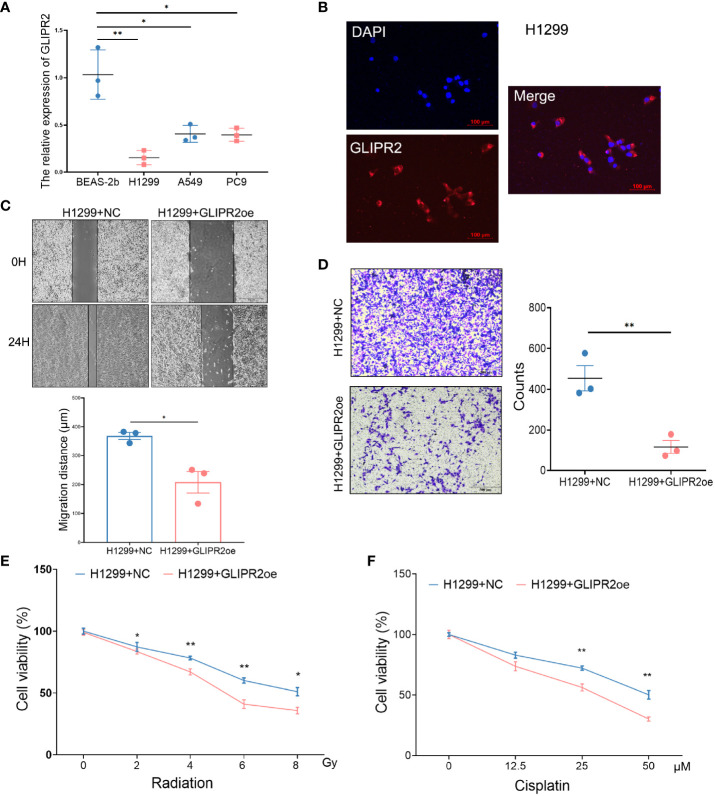
Functional experiments of GLIPR2 *in vitro*. **(A)** Distinct LUAD cell lines and normal lung epithelial cell lines exhibit varying patterns of GLIPR2 expression (n = 3). **(B)** Immunofluorescence analysis reveals predominant cytoplasmic distribution of GLIPR2 in H1299 cell, scale bar = 100 μm. **(C, D)** GLIPR2 block cell migration (C, n = 3) and invasion (D, n = 3) in H1299 cell line. **(E, F)** Elevated expression of GLIPR2 in H1299 cells enhances both radiosensitivity (n = 4) and chemosensitivity (n = 4). *P < 0.05, **P < 0.01.

In order to substantiate the functional implications of GLIPR2, a series of *in vitro* experiments were conducted. The mRNA expression of GLIPR2 exhibited a noteworthy elevation in normal lung epithelial cells in comparison to LUAD cell lines (**Figure 9A**). The H1299 cell line, characterized by the lowest GLIPR2 expression, was subsequently selected for further investigations. Immunofluorescence staining demonstrated a diffuse cytosolic distribution of GLIPR2 in H1299 cells (**Figure 9B**). Functional gain experiments involving GLIPR2 overexpression in the H1299 cell line revealed a pronounced inhibition of migration (**Figure 9C**) and invasion (**Figure 9D**). Moreover, augmentation of GLIPR2 attenuated radiotherapy resistance (**Figure 9E**) and concurrently induced susceptibility to chemotherapy (**Figure 9F**) in H1299 cells.

Collectively, the culmination of these findings collectively underscores the significant implications of GLIPR2 in the domain of LUAD. These observations accentuate its potential as both a prognostic and predictive marker, particularly in the context of radiotherapy to predict treatment responses. The multifaceted facets of GLIPR2 impact on therapeutic outcomes highlight its promise for translational applications within the clinical management of post-radiotherapy LUAD patients.

## 4 Discussion

In this present investigation, we employed a comprehensive array of bioinformatics analytical methodologies to investigate the potential implications of the GLIPR2 gene in cancer progression. Our findings reveal a marked reduction in GLIPR2 expression, strongly associated with the clinical stage across a diverse spectrum of malignancies Additionally, ROC curve analysis highlights the latent potential of GLIPR2 as a promising diagnostic biomarker across various cancer subtypes, including but not limited to CESC, CHOL, COAD, CEAD, KICH, LUAD, and LUSC.

Genetic mutations, particularly when coupled with DNA methylation alterations, exert profound influences on tumorigenesis (32, 33). Our study observed a significant downregulation of GLIPR2 expression in most types of cancer, accompanied by a simultaneous increase in the mutation rates associated with methylation events. This dual phenomenon suggests a potential role of GLIPR2 in cancer pathogenesis and highlights the intricate relationship between gene expression regulation and epigenetic modifications. The concurrent rise in methylation mutation rates further underscores the intricate epigenetic landscape in cancer progression. Methylation alterations, particularly in the promoter regions of tumor suppressor genes, can lead to transcriptional silencing and contribute to tumorigenesis. The observed correlation between GLIPR2 downregulation and increased methylation mutation rates suggests a potential mechanism through which cancer cells may evade the tumor-suppressive effects of GLIPR2. The identification of this association opens avenues for exploring GLIPR2 as a potential therapeutic target. Strategies aimed at reversing or mitigating the methylation alterations linked to GLIPR2 downregulation could represent novel therapeutic interventions in cancer treatment. Genetic mutations involve enduring alterations in the DNA sequence, modifying gene functionalities, dysregulation, and anomalous activations or inactivation, thereby contributing to tumor inception and progression (34–36). Such mutations include point mutations, insertions, deletions, and inversions, leading to modifications in the protein structure and function encoded by the genes (37). Conversely, DNA methylation is an epigenetic modification involving the addition of methyl groups to DNA molecules (38). While DNA methylation regulates normative cells, aberrant patterns are frequently encountered in cancerous cells. In malignancies, methylation is frequently associated with gene silencing, precipitating the subdued expression of normative genes (39). These methylation alterations impinge upon tumor suppressor genes and oncogenes, influencing cellular proliferation, survival, and invasive propensities. Within the intricate milieu of diverse TME, a total of thirty-four mutations, characterized by an indeterminate degree of significance, were delineated via a comprehensive analysis of GLIPR2. Particularly salient is the observation that amidst this collection of mutations, a conspicuous elevation in the levels of GLIPR2 methylation was discerned in LUAD, whereas a converse pattern was manifest in LUSC. Galvanized by these discernments, the designation of NSCLC as the focal point for subsequent validation endeavors was judiciously warranted.

LUAD and LUSC represent prominent subtypes of lung cancer, exhibiting both shared characteristics and distinguishing features. Emerging within pulmonary tissues, these subtypes diverge in terms of their cellular origins, molecular profiles, and clinical presentations (40, 41). LUAD originates from lung glandular cells, which contribute to mucus and other secretions, whereas LUSC arises from lung squamous epithelial cells characterized by their flattened morphology. Mutations in genes such as epidermal growth factor receptor (EGFR) and anaplastic lymphoma kinase (ALK) are frequently implicated in LUAD (42), while the p53 gene mutations are prevalent in LUSC (43). In our study, upon subjecting NSCLC tissues to rigorous *in vitro* experimentation, a pronounced down-regulation in the expression of GLIPR2 became evident. Strikingly, IHC scores associated with GLIPR2 in stage III and IV LUAD instances displayed a statistically significant augmentation compared to stage I and II. In contrast, such a trend was not replicated within the context of LUSC specimens. Furthermore, a focused scrutiny of LUAD revealed an intensified manifestation of GLIPR2, correlating with a conspicuously improved prognosis. Conversely, no overt discordance in consequential survival outcomes emerged with respect to GLIPR2 expression within the purview of LUSC. Particularly pivotal is the observation that among LUAD patients subjected to post-radiotherapy, heightened levels of GLIPR2^+^ infiltration correlated with an augmented frequency of expression in radiation-sensitive cases, in contradistinction to their radiation-resistant counterparts. Collectively, these findings collectively posit the plausible implication of GLIPR2 in the mechanistic underpinnings governing the genesis and pathological progression of LUAD.

GLIPR2 was first discovered within the human genome, displaying a broad expression profile. Notably, investigations into GLIPR2’s interactions have uncovered a Tat-beclin 1 peptide derived from beclin 1, demonstrating autophagy-inducing properties with potential therapeutic applications, particularly in the context of HIV-1 Nef interaction (44). In the realm of colorectal cancer (CRC), GLIPR2’s correlation with glycolysis-related genes and its involvement in epithelial-to-mesenchymal transition (EMT) suggested its pivotal role in tumor progression (45). Furthermore, in hepatocellular carcinoma (HCC), GLIPR2’s upregulation in hypoxia contributes to migration and invasion through the hypoxia/GLIPR-2/EMT axis (46). In our study, we confirmed GLIPR’ broad expression profile, notably elevated in lung, prostate, colon, and rectum, while comparatively diminished in cerebral cortex, parathyroid gland, epididymis, and soft tissues (10). Additionally, our investigation extends GLIPR2’s relevance to cancer immunity, emphasizing its role in the TME. Our results aligned with previous studies, revealing positive correlations between GLIPR2 expression and immune cell content in the TME across various cancers (47). In particular, our analysis, consistent with ESTIMATE analysis, establishes a positive association between GLIPR2 and the infiltration levels of various immune cells in the TME of LUAD, including CD4^+^ T cells, CD8^+^ T cells, MDSCs, NKT cells, Tregs, B cells, myeloid dendritic cells, monocytes, and macrophage M2. These findings position GLIPR2 as a potential biomarker for LUAD immunotherapy, intricately linked to the extent of immune cell infiltration. Collectively, these studies, including our own, underscore GLIPR2’s versatile roles in autophagy, cancer, and immune response, emphasizing its significance as a diagnostic marker and therapeutic target across diverse pathological conditions.

Our study explores the multifaceted role of GLIPR2 in NSCLC, leveraging insights from a real-world cohort at Nantong Tumor Hospital. The progressive reduction in GLIPR2 expression with LUAD tumor progression suggests its potential involvement in underlying mechanisms driving LUAD development. Notably, GLIPR2’s prognostic relevance is histotype-specific, exhibiting significance in LUAD but not in LUSC, indicative of distinct molecular pathways governing these NSCLC subtypes. In-depth analyses of treatment cohorts, particularly post-radiotherapy cases, establish GLIPR2 as a prognostic indicator in LUAD. Furthermore, our *in vitro* experiments, while acknowledging their limitations in capturing the tumor immune microenvironment complexity, demonstrate GLIPR2 augmentation sensitizing tumor cells to radiotherapy. This aligns with clinical findings, emphasizing GLIPR2’s potential as a predictive biomarker for radiotherapy response. Discrepancies between clinical and *in vitro* results are discussed within the clinical complexity of chemotherapy, underscoring the challenges of interpreting *in vitro* findings in the context of combination therapy and varied pharmacological mechanisms. These insights necessitate cautious interpretation of *in vitro* results and stress the importance of clinical validation. Looking ahead, these findings lay the foundation for future investigations into the underlying molecular mechanisms driving observed correlations. Mechanistic studies and analyses of larger patient cohorts will provide deeper insights into GLIPR2’s functional relevance in NSCLC pathobiology, potentially guiding personalized therapeutic strategies.

In conclusion, this study utilized diverse bioinformatics approaches to comprehensively investigate the roles of GLIPR2 in NSCLC, highlighting its potential implications in cancer development, diagnosis, mutation, methylation, and immune infiltration. These findings not only provide novel perspectives on our understanding of cancer biology but also offer crucial leads for early LUAD diagnosis and therapeutic target development.

## Data availability statement

The original contributions presented in the study are included in the article/**Supplementary Material**. Further inquiries can be directed to the corresponding authors.

## Ethics statement

The studies involving humans were approved by the clinical archives of the Affiliated Tumor Hospital of Nantong University and Nantong Third People’s Hospital. The studies were conducted in accordance with the local legislation and institutional requirements. The participants provided their written informed consent to participate in this study.

## Author contributions

WL: Conceptualization, Supervision, Visualization, Writing – original draft, Writing – review & editing. SZ: Formal analysis, Investigation, Validation, Writing – review & editing. CG: Funding acquisition, Supervision, Writing – review & editing. HZ: Funding acquisition, Supervision, Writing – review & editing. LY: Conceptualization, Formal analysis, Supervision, Visualization, Writing – review & editing.

## Supplementary material

Supplementary Figure 1 Mutation patterns of GLIPR2 across pan-cancer spectrum. **(A)** Representation of GLIPR2 genetic alterations in various cancer types. **(B)** Illustration of GLIPR2 variants of uncertain significance (VUS) across diverse tumor contexts.

Supplementary Figure 2 Promoter methylation patterns of GLIPR2 across pan-cancer types.

Supplementary Figure 3 Correlation of GLIPR2 expression levels with related immunomodulatory genes.

Supplementary Figure 4 Diagnostic potential of GLIPR2 across pan-cancer types. **(A–X)** Receiver operating characteristic (ROC) curves depicting the performance of GLIPR2 in terms of its diagnostic value for PCPG, BLCA, BRCA, CESC, CHOL, COAD, CEAD, ESCA, GBM, HNSC, KICH, KIRC, KIRP, HCC, LUAD, LUSC, SARC, PAAD, PRAD, STAD, THCA, THYM, UCEC, and OSCC.

Supplementary Figure 5 Overall survival (OS) curves according to GLIPR2^+^ infiltration level of patients in TCGA database.

Supplementary Figure 6 The intensity and positive cells of GLIPR2 among CESC, LUAD and LUSC in Nantong Third People’ Hospital cohort (n = 6, **P* < 0.05).
