# Supplementary figures and images for: GLIPR2: a potential biomarker and therapeutic target unveiled – Insights from extensive pan-cancer analyses, with a spotlight on lung adenocarcinoma
Source: Front Immunol. 2024 Feb 26;15:1280525. doi: 10.3389/fimmu.2024.1280525 (PMC10929020; doi:10.3389/fimmu.2024.1280525)

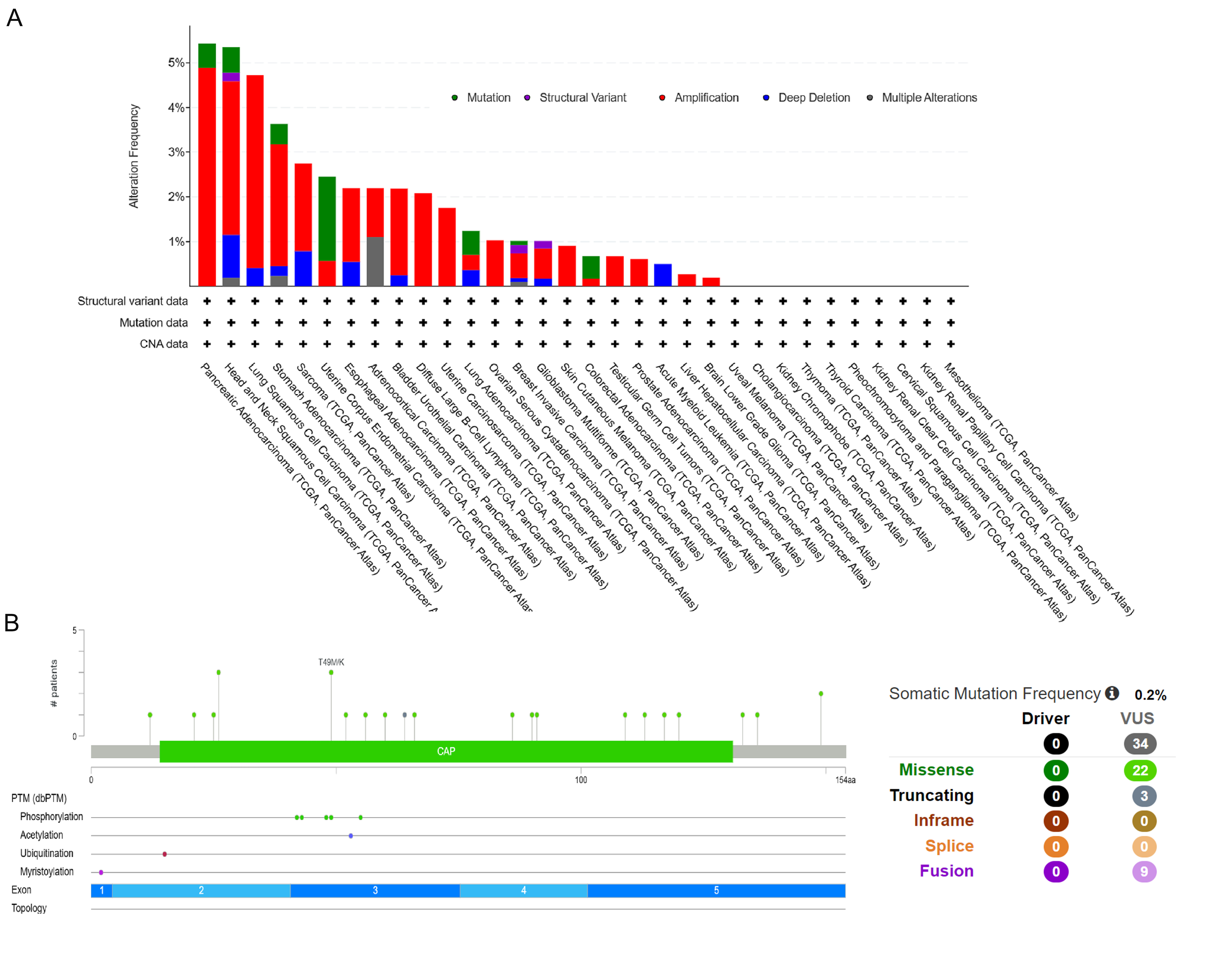

Supplement: Supplementary Figure 1 — Mutation patterns of GLIPR2 across pan-cancer spectrum. (A) Representation of GLIPR2 genetic alterations in various cancer types. (B) Illustration of GLIPR2 variants of uncertain significance (VUS) across diverse tumor contexts. [file Image_1.tif]

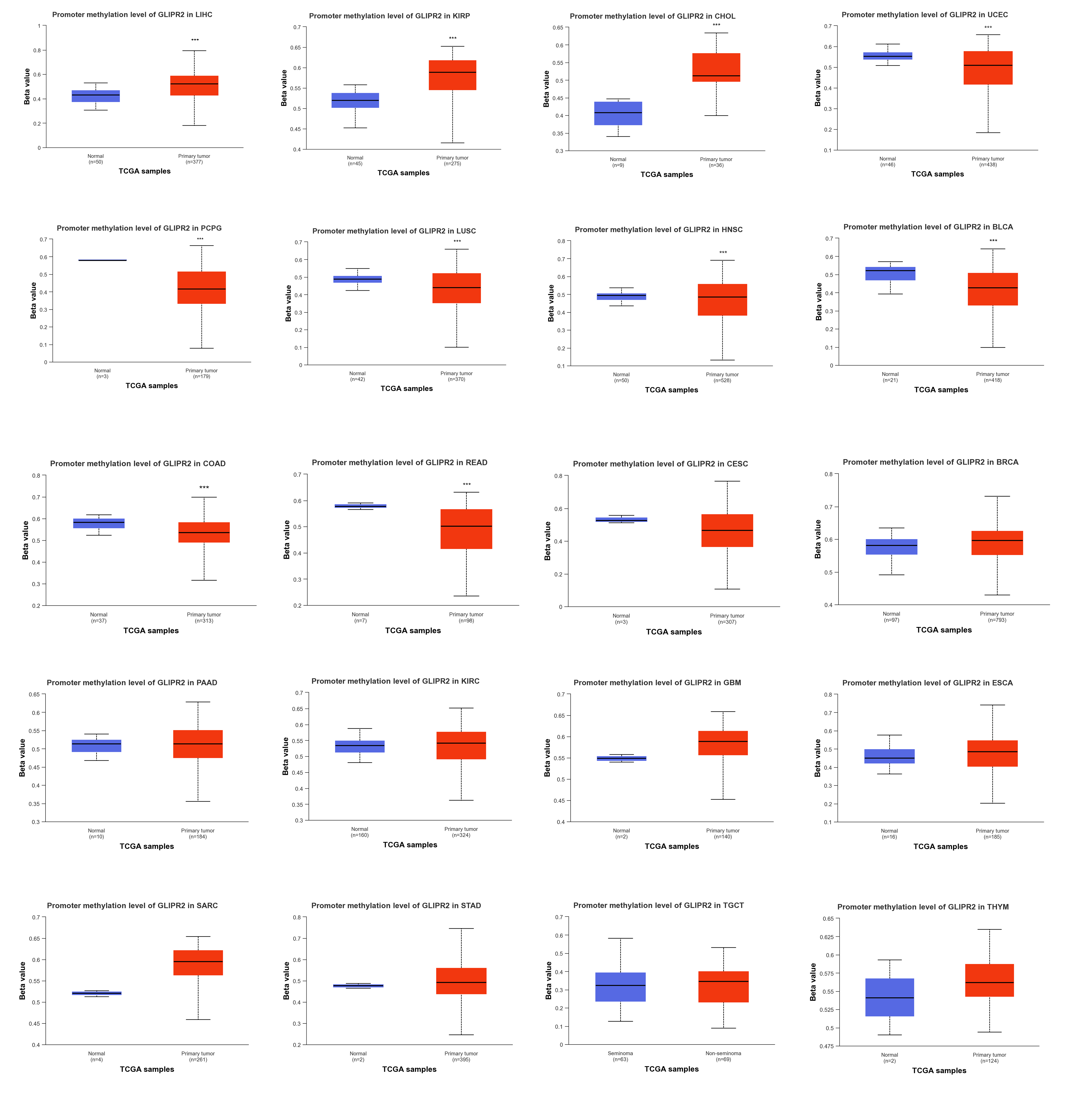

Supplement: Supplementary Figure 2 — Promoter methylation patterns of GLIPR2 across pan-cancer types. [file Image_2.tif]

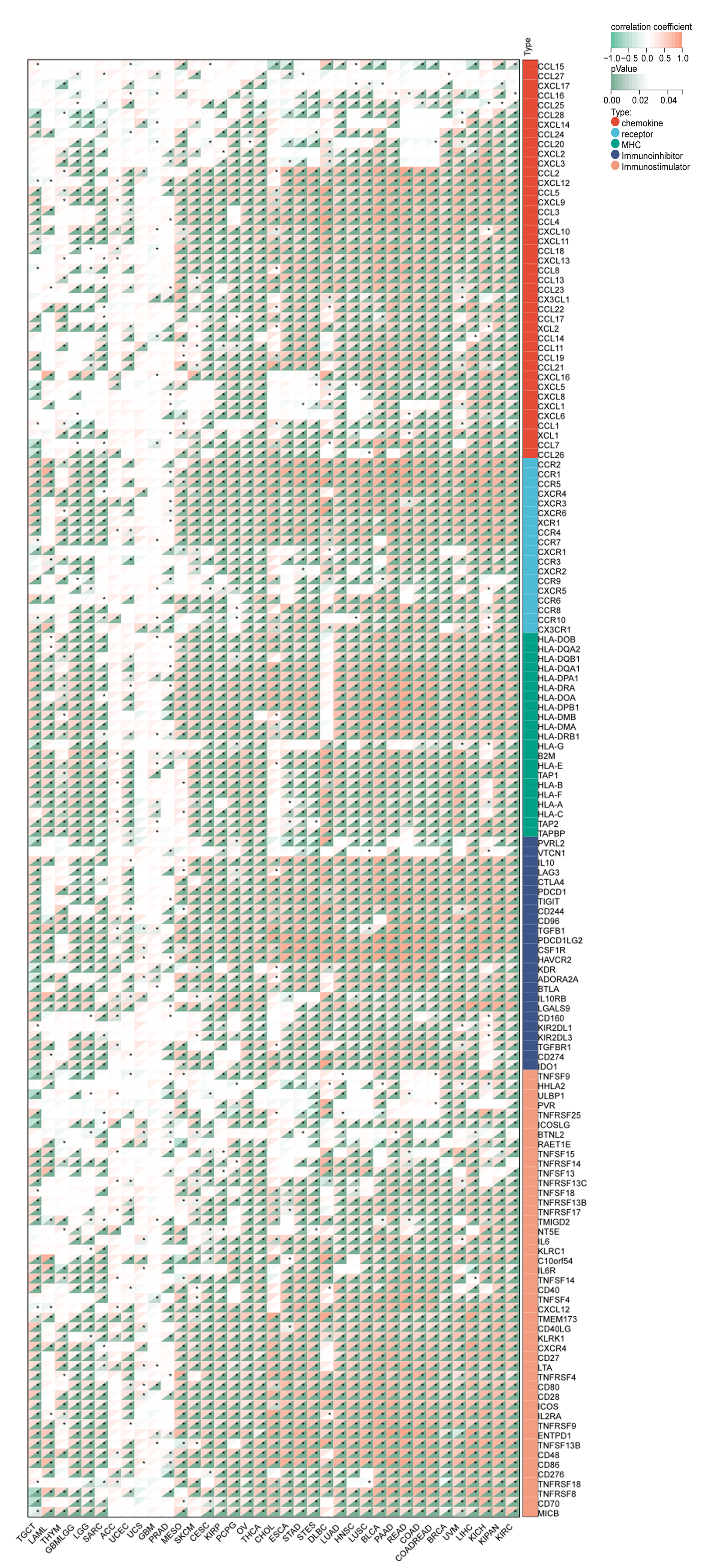

Supplement: Supplementary Figure 3 — Correlation of GLIPR2 expression levels with related immunomodulatory genes. [file Image_3.tif]

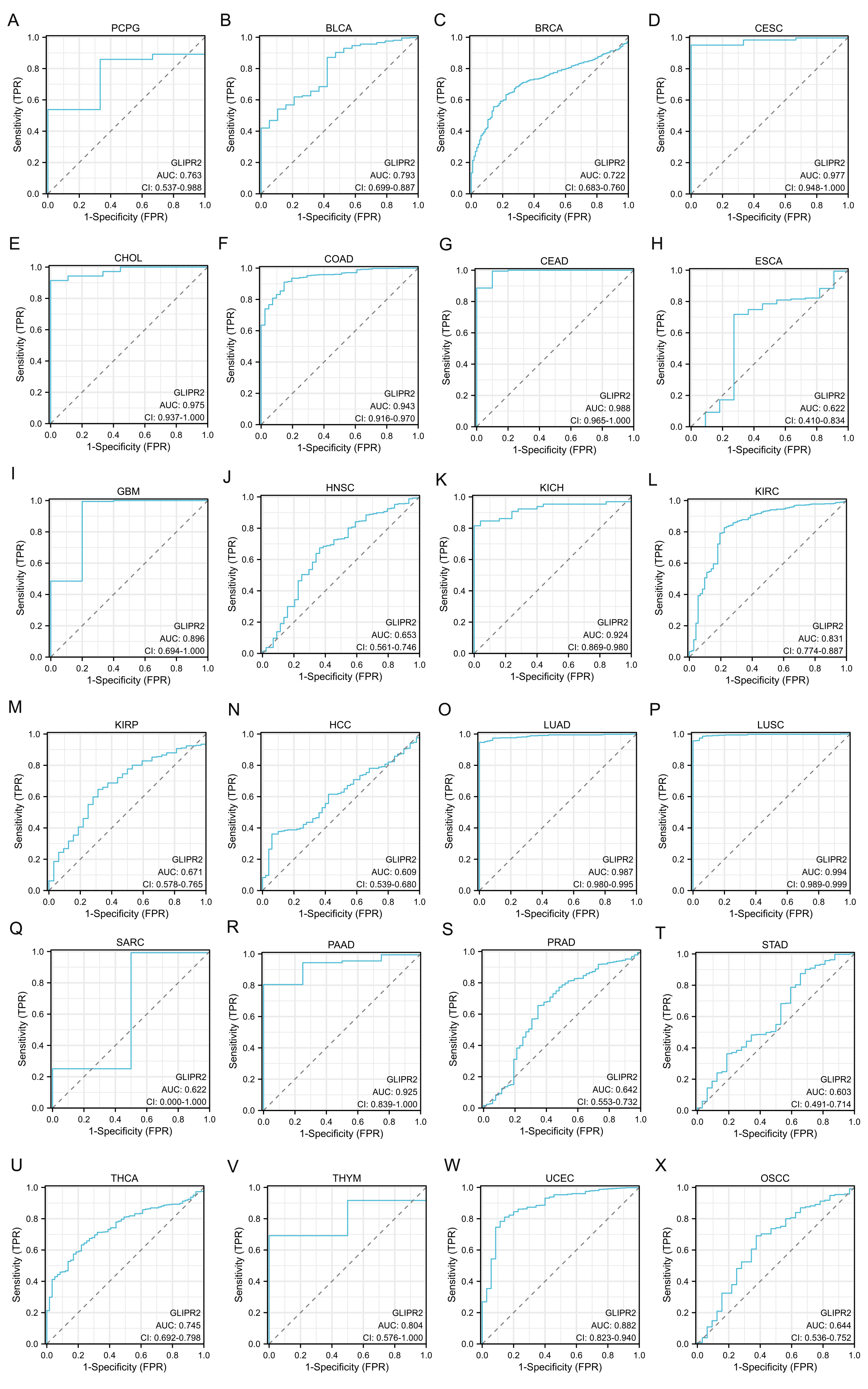

Supplement: Supplementary Figure 4 — Diagnostic potential of GLIPR2 across pan-cancer types. (A–X) Receiver operating characteristic (ROC) curves depicting the performance of GLIPR2 in terms of its diagnostic value for PCPG, BLCA, BRCA, CESC, CHOL, COAD, CEAD, ESCA, GBM, HNSC, KICH, KIRC, KIRP, HCC, LUAD, LUSC, SARC, PAAD, PRAD, STAD, THCA, THYM, UCEC, and OSCC. [file Image_4.tif]

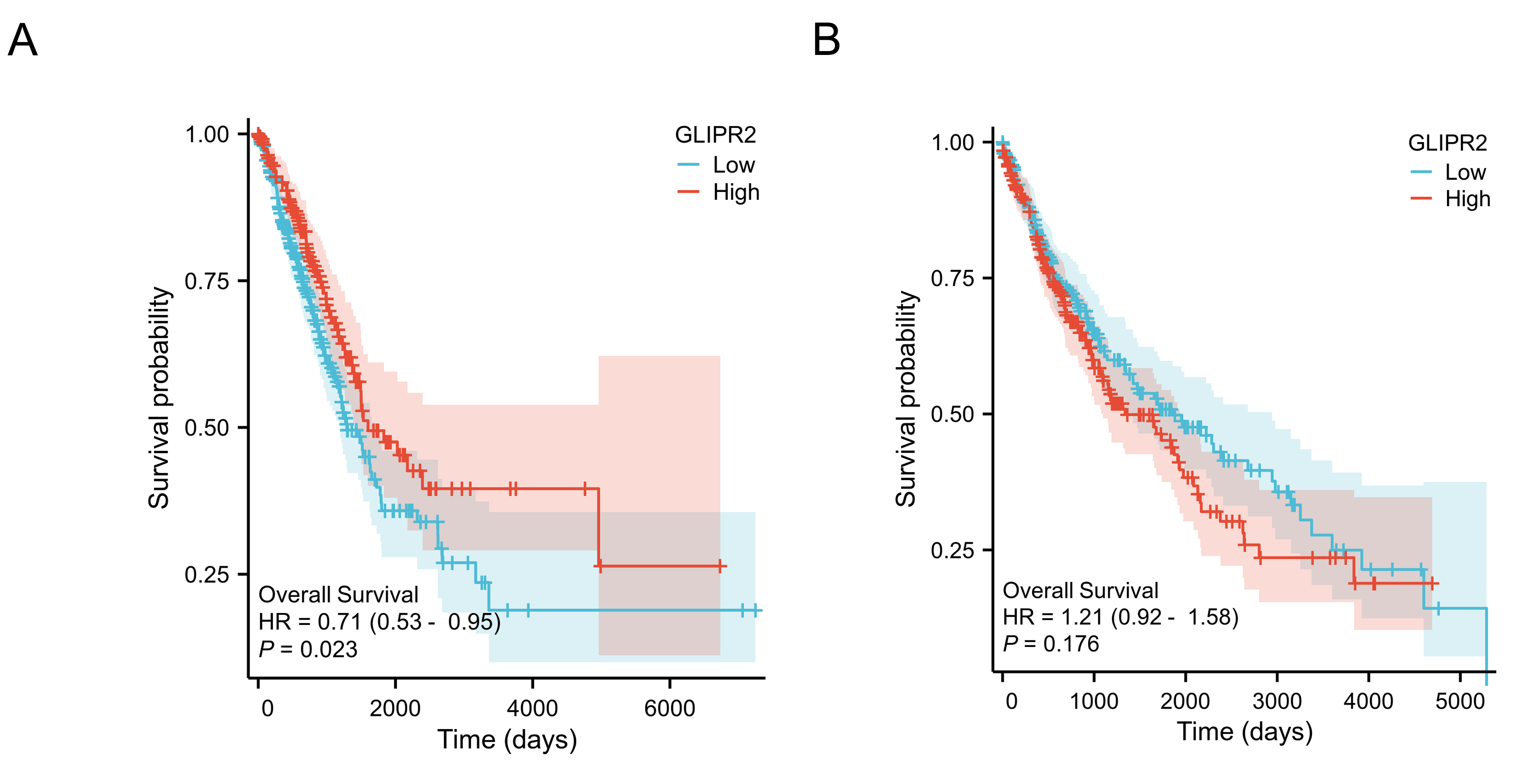

Supplement: Supplementary Figure 5 — Overall survival (OS) curves according to GLIPR2+ infiltration level of patients in TCGA database. [file Image_5.tif]

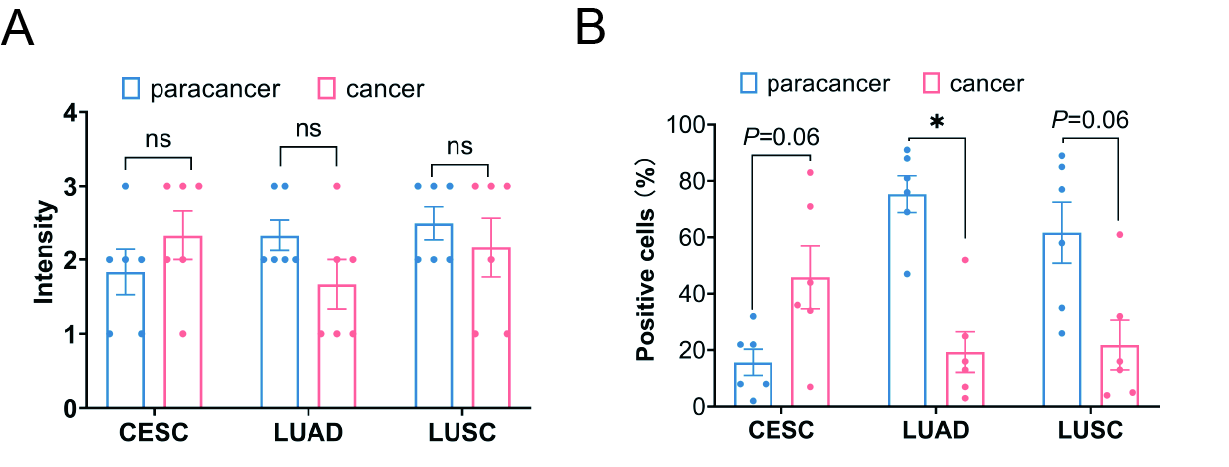

Supplement: Supplementary Figure 6 — The intensity and positive cells of GLIPR2 among CESC, LUAD and LUSC in Nantong Third People’ Hospital cohort (n = 6, *P < 0.05). [file Image_6.tif]
